# Kinetics and Molecular Docking Studies of 6-Formyl Umbelliferone Isolated from *Angelica decursiva* as an Inhibitor of Cholinesterase and BACE1

**DOI:** 10.3390/molecules22101604

**Published:** 2017-09-24

**Authors:** Md Yousof Ali, Su Hui Seong, Machireddy Rajeshkumar Reddy, Sung Yong Seo, Jae Sue Choi, Hyun Ah Jung

**Affiliations:** 1 Department of Food and Life Science, Pukyong National University, Busan 48513, Korea; yousufbge@gmail.com (M.Y.A.); seongsuhui@naver.com (S.H.S.); 2 Department of Chemistry, Pukyong National University, Busan 48513, Korea; rajeshr.chem@gmail.com (M.R.R.); syseo@pknu.ac.kr (S.Y.S.); 3 Department of Food Science and Human Nutrition, Chonbuk National University, Jeonju 54896, Korea

**Keywords:** *Angelica decursiva*, 6-formyl umbelliferone, coumarins, BACE1, cholinesterases, molecular docking

## Abstract

Coumarins, which have low toxicity, are present in some natural foods, and are used in various herbal remedies, have attracted interest in recent years because of their potential medicinal properties. In this study, we report the isolation of two natural coumarins, namely umbelliferone (**1**) and 6-formyl umbelliferone (**2**), from *Angelica decursiva*, and the synthesis of 8-formyl umbelliferone (**3**) from **1**. We investigated the anti-Alzheimer disease (anti-AD) potential of these coumarins by assessing their ability to inhibit acetylcholinesterase (AChE), butyrylcholinesterase (BChE), and β-site amyloid precursor protein (APP) cleaving enzyme 1 (BACE1). Among these coumarins, **2** exhibited poor inhibitory activity against AChE and BChE, and modest activity against BACE1. Structure–activity relationship analysis showed that **2** has an aldehyde group at the C-6 position, and exhibited strong anti-AD activity, whereas the presence or absence of an aldehyde group at the C-8 position reduced the anti-AD activity of **3** and **1**, respectively. In addition, **2** exhibited concentration-dependent inhibition of peroxynitrite-mediated protein tyrosine nitration. A kinetic study revealed that **2** and **3** non-competitively inhibited BACE1. To confirm enzyme inhibition, we predicted the 3D structures of AChE and BACE1, and used AutoDock 4.2 to simulate binding of coumarins to these enzymes. The blind docking studies demonstrated that these molecules could interact with both the catalytic active sites and peripheral anionic sites of AChE and BACE1. Together, our results indicate that **2** has an interesting inhibitory activity in vitro, and can be used in further studies to develop therapeutic modalities for the treatment of AD.

## 1. Introduction

Alzheimer’s disease (AD) is a progressive neurodegenerative disorder with characteristic features of memory impairment, cognitive dysfunction, behavior disturbances, and deficits in activity of daily living [1]. The increasing prevalence of AD has resulted in more interest in identifying risk factors associated with the development of AD [2]. AD poses a huge economic burden worldwide, because there is no cure for this disease, which becomes progressively worse and eventually leads to death [3]. Two major hypotheses have been proposed regarding the molecular mechanism of AD pathogenesis: the cholinergic hypothesis and the amyloid cascade hypothesis [4]. However, these two major hypotheses cannot explain all pathological pathways of AD. Numerous recent studies have reported correlations between AD, inflammation, and oxidative stress [5,6,7]. AD has been reported to be highly associated with cellular oxidative stress, including augmentation of protein oxidation, protein nitration, and accumulation of amyloid-β peptide (Aβ) [6]. In vivo formation of ONOO^−^ has been implicated in Aβ formation and accumulation, with high levels of Aβ also augmenting ONOO^−^ generation in brains of AD patients [8,9,10]. Simultaneous studies on cholinesterase (ChEs), Aβ accumulation inhibitory effects, and antioxidant effects are considered integral to the development of promising anti-AD agents. Several of the BACE1 (AZD3293, E2609, MK-8931, RG7129 10 and LY2886721) and cholinesterase (donepezil, tacrine, rivastigmine, and galantamine) inhibitors currently in use have adverse side-effects, including gastrointestinal disturbances, such as nausea, vomiting, and diarrhea; they also have bioavailability issues [11,12,13].

For these reasons, there is growing scientific interest in identifying natural sources of AChE, BChE, and BACE1 inhibitors with safer profiles.

6-Formyl umbelliferone (**2**), an uncommon coumarin derivative in nature, has been isolated from *Angelica decursiva*, a perennial herb distributed on hillsides, grasslands, and sparse forests within China, Japan, and Korea. This plant has long been used in traditional Korean medicine as an antitussive, analgesic, antipyretic, and cough remedy. In traditional Chinese medicine, *A*. *decursiva* is used as to treat thick phlegm, asthma, and upper respiratory tract infections [14,15,16,17]. We previously reported that coumarins from *A*. *decursiva* have promising anti-AD, anti-diabetic, antioxidant, and anti-inflammatory activity [15,16,18,19,20,21]. Coumarin and its derivatives have been reported to show a wide range of pharmacological activity, such as anticoagulant, estrogenic, dermal photosensitizing, vasodilator, molluscicidal, anthelmintic, sedative, hypnotic, analgesic, hypothermic, antimicrobial, anti-inflammatory, antifungal, and antiulcer activity [22]. Caffieri et al. [23] reported the apoptogenic effects of 6-formyl umbelliferone on mitochondria. Other than their study, the biological properties of **2** have not been further investigated.

In the present study, we evaluated the anti-AD potential of coumarins by assessing their ability to inhibit AChE, BChE, BACE1, and ONOO^−^-mediated tyrosine nitration. Because there is currently no detailed information regarding 3D molecular interactions between these coumarins and BACE1 and AChE, we performed molecular docking analyses and detailed enzyme kinetic analysis to investigate the potential of coumarins as potent anti-AD drug candidates.

## 2. Results

### 2.1. Inhibitory Activity of Coumarins against AChE, BChE, and BACE1

**Figure 1:**
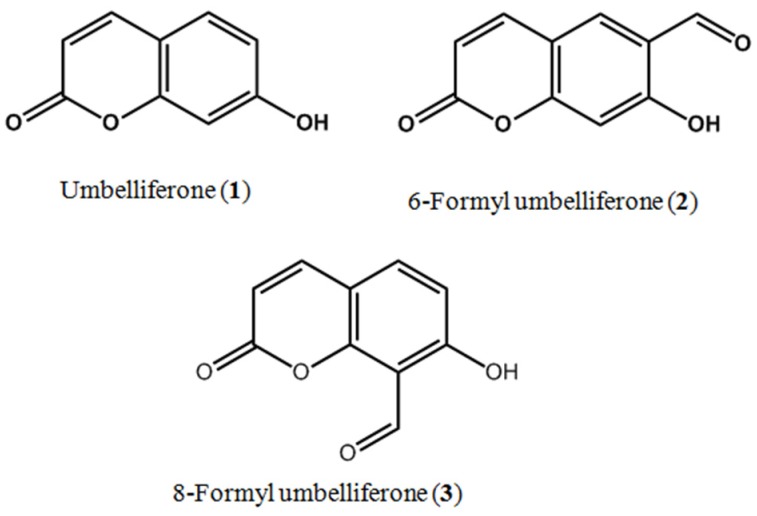
Chemical structures of umbelliferone, 6-formyl umbelliferone, and 8-formyl umbelliferone.

**Figure 2:**
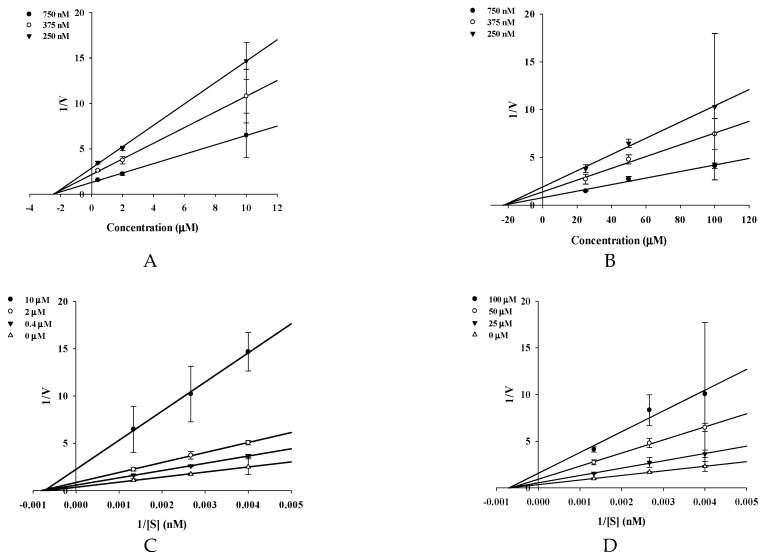
Dixon plots for BACE1 inhibition of **2** (**A**) and **3** (**B**) were tested in the presence of different concentrations of substrate: 750 nM (●), 375 nM (○), 250 nM (▼). Lineweaver–Burk plots for BACE1 inhibition of compounds were analyzed in the presence of different concentrations of sample as follows: 0 µM (Δ), 0.4 µM (▼), 2 µM (○), 10 µM (●) for **2** (**C**); 0 µM (Δ), 25 µM (▼), 50 µM (○), 100 µM (●) for **3** (**D**).

To evaluate the potential anti-AD activity of coumarins **1**–**3** (Figure 1), their inhibitory potential against electric eel AChE, horse serum BChE, and human recombinant BACE1 was evaluated (Table 1). As shown in Figures S1–S3, ChEs and BACE1 were inhibited by **1**–**3** in a dose-dependent manner. Especially, **2** exhibited potent BACE1 (Figure S3B) inhibitory activity, with an IC_50_ value of 1.31 ± 0.01 µM. However, **1** and **3** were weak BACE1 inhibitors, with respective IC_50_ values of 168.54 ± 2.17 and 39.82 ± 0.31 µM. In addition, **2** showed higher inhibitory activity against AChE and BChE than **3** and **1** (IC_50_ values of 16.70 ± 1.62 (**2**), 19.13 ± 0.57 (**3**), and 105.48 ± 0.57 (**1**) µM for AChE; 27.90 ± 3.43 (**2**), 87.67 ± 0.48 (**3**), and 90.14 ± 0.02 (**1**) µM for BChE). The selective index (SI) of **1**, **2**, and **3,** calculated from IC_50_ of BChE/AChE, were 0.85, 1.67, and 4.58, respectively (Table 1). However, **2** was less effective than the positive control, berberine (IC_50_ values of 0.14 ± 0.08 for AChE, and 9.81 ± 0.35 µM for BChE). To determine the mode of BACE1 inhibition exerted by **2** and **3**, inhibition kinetics were investigated using Lineweaver–Burk plots and Dixon plots at different substrate and inhibitor concentrations. The Lineweaver–Burk plots showed that **2** and **3** exhibited noncompetitive-type inhibition against BACE1, because each of the lines intersected on the *x*-axis, and the *y*-intercept increased with increasing inhibitor concentration (Figure 2A,B). In the Dixon plot, the values of the *x*-axis represent an inhibition constant (*K_i_*). As shown in Figure 2C,D, the *K_i_* values of **2** and **3** were 2.27 and 22.2 µM, respectively.

### 2.2. Molecular-Docking Study of the Inhibition of AChE by Coumarins

**Figure 3:**
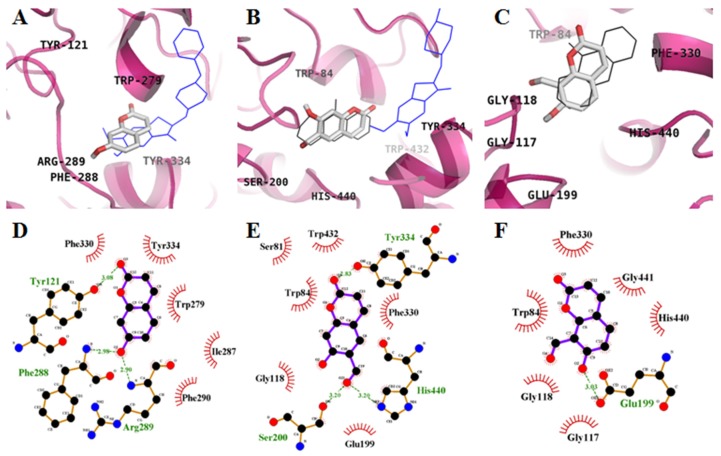
Molecular docking models of AChE inhibition by **1** (**A**), **2** (**B**), and **3** (**C**) with THA (black line) and donepezil (blue line). Ligand interaction diagram of AChE inhibition of **1** (**D**), **2** (**E**), **3** (**F**). Dashed lines indicate hydrogen bonds. Carbons are in black, nitrogens in blue, and oxygens in red. Figures were generated using PyMOL (v1.7.4, Schrödinger, LLC, Cambridge, MA, USA) and Ligplot+ (v1.4.5, European Bioinformatics Institute, London, England).

Molecular-modeling studies were performed to gain insights into the binding of target coumarins to AChE. AChE-**1**, **2**, and **3** inhibitor complexes had binding energies of −6.3, −8.3, and −8.0 kcal/mol, respectively (Table 2). As shown in Figure 3A,D, **1** formed three hydrogen bonds with the interacting residues Tyr121, Phe288, and Arg289. Phe288 and Arg289 were involved in strong hydrogen bonding interactions with the hydroxyl group at position C-7, and Tyr121 was also involved in hydrogen bonding interactions with the ketone group at position C-2. In particular, important peripheral anionic site (PAS) residues (Trp279 and Tyr334) of AChE were involved in hydrophobic interactions with **1**. Interestingly, **2** docked into both the catalytic anionic site (CAS) and PAS of AChE (Figure 3B). The binding site for **2** was formed by residues Ser200, Tyr334, His440, Ser81, Trp84, Gly118, Glu199, Phe330, and Trp432 (Figure 3E). An aldehyde group at position C-6 formed two strong hydrogen bonds with the catalytic triad, Ser200 and His400, whereas the ketone group at position C-2 interacted with Tyr334, which is involved in PAS, via a hydrogen bonding interaction. However, **3** docked into same catalytic pocket as tacrine (THA) in AChE (Figure 3C). The docking analysis predicted that the hydroxyl group at position C-7 of **3** formed a hydrogen bond with the Glu199 residue. In addition, residues Trp84, Gly117, Gly118, Phe330, His440, and Gly441 were found to be involved in hydrophobic interactions with the coumarin (Figure 3F). These results indicated that the notable inhibitory activity of **2** might be due to interaction with both important catalytic and allosteric residues of AChE.

### 2.3. Molecular-Docking Study of the Inhibition of BACE1 by Coumarins

**Figure 4:**
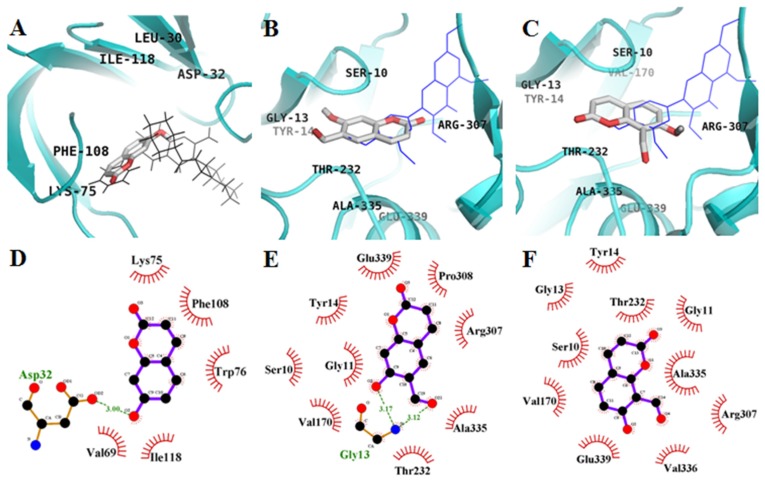
Molecular docking models of BACE1 inhibition of **1** (**A**), **2** (**B**), and **3** (**C**) with QUD (black line) and PMF (blue line). Ligand interaction diagram of BACE1 inhibition of **1** (**D**), **2** (**E**), **3** (**F**). Dashed lines indicate hydrogen bonds. Carbons are in black, nitrogens in blue, and oxygens in red. Figures were generated using PyMOL and Ligplot+.

Molecular docking models of coumarins, 2-amino-3-{(1*R*)-1-cyclohexyl-2-[(cyclohexylcarbonyl) amino]ethyl}-6-phenoxyquinazolin-3-ium (QUD), and 3,5,7,3′,4′-pentamethoxyflavone (PMF) are illustrated in Figure 4 and Table 3. QUD is a non-peptic catalytic BACE1 ligand reported in the PDB, while PMF is a previously reported noncompetitive inhibitor of BACE1 [24]. As illustrated in Figure 4A,D, inspection of binding interactions in the active site indicated that the hydroxyl group of **1** interacts with the catalytic residue Asp32 of the enzyme via a hydrogen bonding interaction. In addition, hydrophobic interactions between umbelliferone and Lys75, Trp76, Val69, Phe108, and Ile118 were observed with a binding energy of −5.4 kcal/mol. In contrast to **1**, the top binding sites of **2** and **3** were distant from the catalytic site, but similar to those of PMF. As shown in Figure 4B,E, Gly13 of BACE1 formed two hydrogen bonds with the hydroxyl and formyl groups of **2** with a binding energy of −7.2 kcal/mol. Moreover, hydrophobic interactions between **2** and Ser10, Gly11, Tyr14, Val170, Thr232, Arg307, Pro308, Ala335, and Glu339 of BACE1 also seemed important for binding to the allosteric site, based on inspection of our AutoDock 4.2 model. The molecular docking model of **3** is illustrated in Figure 4C,F. In the **3**–BACE1 complex, Ser10, Gly11, Gly13, Tyr14, Val170, Thr232, Arg307, Ala335, Val336, and Glu339 participated in hydrophobic interactions with **3** without any hydrogen bonding interactions.

### 2.4. Inhibitory Activity of 6-Formyl Umbelliferone (**2**) against ONOO^−^-Mediated Tyrosine Nitration

**Figure 5:**
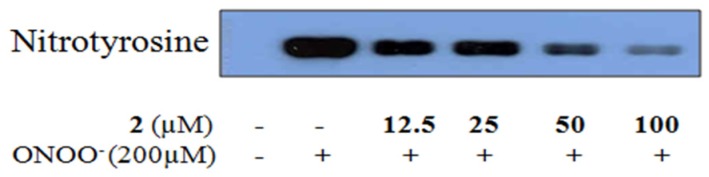
Effect of **2** on nitration of BSA by ONOO^−^. A mixture of the compound and BSA was incubated at 25 °C for 10 min. After incubation of ONOO^−^ at 25 °C for 10 min, reactants were resolved by electrophoresis in a 10% SDS-polyacrylamide gel.

To determine the inhibitory effect of **2** against ONOO^−^-induced tyrosine nitration, Western blot analysis was performed using a 3-nitrotyrosine antibody. **2** exhibited inhibitory effects on ONOO^−^-mediated tyrosine nitration, as shown in Figure 5. Pretreatment with **2** at different concentrations (12.5–100 μM) resulted in significant dose-dependent inhibition of ONOO^−^-mediated tyrosine nitration.

## 3. Discussion

Medicinal plants have long provided a reliable source of new drugs to combat diseases. There have also been new trends in the preparation and marketing of drugs based on medicinal plants. Their scientific and commercial significance appears to be gathering momentum in health-relevant areas. Plant-derived products are carefully standardized, and medicinal plants offer several options to modify the progress and symptoms of AD [25,26].

AD presents as the progressive, inexorable loss of cognitive function associated with the presence of senile plaques in the hippocampal area of the brain. The major hurdle in understanding AD is lack of knowledge regarding the etiology and pathogenesis of selective neuron death. The two most common hypotheses used to explain the pathology of AD are the “cholinergic hypothesis” and “amyloid hypothesis.” The cholinergic hypothesis suggests that AD is caused by a deficiency at the brain level of the cerebral neurotransmitter acetylcholine (ACh), which is hydrolyzed by AChE [27,28]. Similarly, BChE activity increases by 40–90% during progression of AD [29], and BChE inhibition is therefore considered a potentially important aspect of AD treatment. In addition to the AChE and BChE hypotheses, accumulation of amyloid-β peptide (Aβ) in the brain is widely considered to be critically involved in the pathogenesis of AD [30]. Aβ plaques emerge roughly 15 years before the symptoms of AD appear [31]. Once AD develops, the cognitive decline caused by neuronal damage cannot be reversed, even if the Aβ level in the brain is lowered by immunotherapy [32]. Thus, prevention of Aβ accumulation is considered an important part of AD prevention. Aβ is excised from amyloid-β precursor protein (APP) through sequential cleavage by aspartic protease β-secretase 1 (BACE1) [28,33]. Because BACE1 initiates Aβ processing, inhibition of BACE1 activity may be an effective way to prevent Aβ accumulation [34].

In recent years, considerable data has accumulated that indicates that brains with AD are under increased oxidative stress. This stress may have a role in the pathogenesis of neurodegeneration and death due to AD. Oxidative stress refers to undue oxidation of biomolecules, and often leads to cellular damage. Histopathological and experimental evidence support the significant impact of oxidation on the pathogenesis of AD [35,36,37].

Many researchers over the past few decades have focused their efforts on designing ChEs and BACE1 inhibitors. However, efforts to discover naturally occurring ChEs and BACE1 inhibitors have been relatively limited. Several plant-derived ChEs and BACE1 inhibitors, including coumarins, anthraquinones, triterpenoids, lignin, flavonoids, and alkaloids [19,38,39,40,41] have been reported. Most of the previously discovered natural BACE1 and ChEs inhibitors have a low molecular weight, exhibit structural similarities, such as aromatic rings, and may have some adverse side effects. Nonetheless, research into effective agents from natural sources is still in its infancy, and there is a crucial need for better ChEs and BACE1 inhibitors from natural resources.

Coumarins are naturally occurring compounds present in a large number of plants. Coumarin and its derivatives are widespread in nature. Coumarin is a benzopyrone; benzopyrones are compounds composed of benzene rings connected to a pyrone moiety. Dietary exposure to benzopyrones is quite significant, as these compounds are found in fruits, vegetables, seeds, nuts, and higher plants. It is estimated that the average Western diet contains ~1 g/day of mixed benzopyrones [42]. Coumarin-containing higher plant extracts are also widely popular in Chinese medicine. Natural coumarins have recently been reported to show promising anti-diabetic, anti-AD, anti-inflammatory, and antioxidant activity [15,16,17,18,19,20,21,43,44]. Coumarins are also considered a promising group of bioactive compounds, because they exhibit a wide range of biological activity, including antibacterial [45], antioxidant [46], anti-inflammatory, and anticoagulant [47] activity. In this study, 6-formyl umbelliferone (**2**), a benzopyrone-type coumarin, was isolated for the first time from *A. decursiva*, and showed activity against electric eel AChE, horse serum BChE, and human recombinant BACE1. We assumed that the inhibitory activity of **2** was mainly due to the formyl moiety at the C-6 position, therefore, we further synthesized 8-formyl umbelliferone (**3**) to determine structure–activity relationships (SARs). To establish SARs between the three coumarins and the target enzymes, the inhibitory effects of the three coumarins against electric eel AChE, horse serum BChE, and human recombinant BACE1 were investigated. **2** and **3** inhibited AChE and BChE with an IC_50_ range of 16.70–19.13 µM for AChE and 27.90–87.67 µM for BChE, whereas umbelliferone (**1**) showed weak inhibitory activity. In addition, **2** strongly inhibited BACE1 with an IC_50_ of 1.31 µM. Among the molecules investigated, both **2** and **3**, which have a free aldehyde group at the 6 or 8 position, significantly inhibited ChEs and BACE1. A comparison of the three coumarins indicated that the presence of an aldehyde group markedly increased inhibitory activity (Table 1). **1** has a free hydroxyl group at the 7 position, but no aldehyde group. The inhibitory activity of ChEs and BACE1 therefore appears to be largely dependent on the presence of the aldehyde group at the 6 or 8 position.

Further enzyme kinetic studies in the presence of varying substrate and inhibitor concentrations revealed that **2** and **3** are noncompetitive BACE1 inhibitors. We next analyzed the molecular structure of AChE-inhibitors (Figure 3) and BACE1-inhibitors (Figure 4) to determine specific functional groups involved in these interactions.

Computational molecular docking analysis can provide insight into the mechanism underlying active site binding interactions. **2** could interact with both CAS (His440 and Ser200) and PAS (Tyr334) of AChE, whereas **3** bound only to CAS (His440). In the BACE1 docking simulation, we observed multiple hydrogen bonds in the docked BACE1–**2** complex. However, **3** did not form any hydrogen bonds with BACE1, even though it docked in a similar region to **2**. These docking data showed that hydrogen bonds between the coumarins and major active residues of the target enzyme play a crucial role in enzyme inhibition. Moreover, we also confirmed the inhibitory activity of **2** against formation of ONOO^−^ mediated tyrosine nitration by Western blotting.

Taken together, the in vitro results and in silico molecular docking data indicate that **2** has significant potential to inhibit, and may prevent AD by targeting Aβ formation through inhibition of BACE1 and AChE, as well as ONOO^−^-mediated tyrosine nitration.

## 4. Material and Methods

### 4.1. General Experimental Procedures

^1^H- and ^13^C-NMR spectra were measured using a JEOL JUM ECP-400 spectrometer (Jeol, Tokyo, Japan) at 400 MHz for ^1^H-NMR and 100 MHz for ^13^C-NMR, with the compounds dissolved in deuterated dimethyl sulfoxide (DMSO-*d*_6_) and chloroform (CDCl_3_). Column chromatography was carried out using 70–230 mesh silica gel (Merck, Darmstadt, Germany). Thin-layer chromatography (TLC) was performed on pre-coated Merck Kiesel gel 60 F_254_ plates (0.25 mm), and 25% H_2_SO_4_ was used as a spray reagent. All solvents for column chromatography were of reagent grade and were acquired from commercial sources.

### 4.2. Chemicals and Reagents

Electric eel acetylcholinesterase (AChE, EC3.1.1.7), horse serum butyrylcholinesterase (BChE, EC 3.1.1.8), acetylthiocholine iodide (ACh), butyrylthioline chloride (BCh), 5,5′-dithobis [2-nitrobenzoic acid] (DTNB), ethylenediaminetetraacetic acid (EDTA), berberine, and quercetin were purchased from Sigma-Aldrich Co. (St. Louis, MO, USA). The BACE1 FREF assay kit (β-secretase) was purchased from Pan Vera Co. (Madison, WI, USA). ONOO^−^ was purchased from Molecular Probes Cayman (Ann Arbor, MI, USA). All other chemicals and solvents used were purchased from E. Merck, Fluka, or Sigma- Aldrich, unless otherwise stated.

### 4.3. Isolation of Coumarins from A. Decursiva

Powder of whole *A. decursiva* plants was refluxed with methanol (MeOH) for 3 h (3 × 10 L). The total filtrate was then concentrated until dry in vacuo at 40 °C to render the MeOH extract. This extract was suspended in distilled water and then successively partitioned with dichloromethane (CH_2_Cl_2_), ethyl acetate (EtOAc), and *n*-butanol (*n*-BuOH) to yield CH_2_Cl_2_, EtOAc, *n*-BuOH, and H_2_O fractions, respectively, according to Zhao et al. [15]. Initially, the EtOAc fraction was also subjected to silica gel column chromatography using CH_2_Cl_2_–MeOH (10:1→0:1, gradient) to obtain 20 subfractions (F-1 to F-20). Repeated chromatography of subfraction-6 over a silica gel column using CH_2_Cl_2_–MeOH (20:1→0:1, gradient) yielded 6-formyl umbelliferone (**2**) (30 mg) and umbelliferone (**1**) (3.9 g), respectively. The structure of the compounds was confirmed by ^1^H- and ^13^C-NMR spectroscopy and comparison with published data [18,23]. Structures are shown in Figure 1.

*6-Formyl umbelliferone* (**2**). Colorless needles; ^1^H-NMR (400 MHz, DMSO-*d_6_*): δ_H_ 11.7 (1H, brs, -OH), 10.24 (1H, s,-CHO), 8.03 (1H, s, H-5), 8.04 (1H, d, *J* = 9.6 Hz, H-4), 6.84 (1H, s, H-8), 6.32 (1H, d, *J* = 9.6 Hz, H-3); ^13^C-NMR (100 MHz, DMSO-*d_6_*): δ_C_ 189.2 (-CHO), 163.4 (C-7), 159.5 (C-2), 158.7 (C-9), 144.4 (C-4), 130.0 (C-5), 120.4 (C-6), 113.1 (C-3), 111.96 (C-10), 103.4 (C-8).

### 4.4. Synthesis of 8-Formyl Umbelliferone

8-Formyl umbelliferone (**3**) was prepared from umbelliferone (2.0 g, 9.8 mmol) and urotropine (2.06 g, 14.69 mmol) in an ice-bath, as described by Qiao et al. [48], and purified by recrystallization and Si gel column chromatography (*n*-hexane/EtOAc = 10:1, *v*/*v*) to yield **3** as a light yellow powder (0.30 g, yield 15%).

*8-Formyl umbelliferone* (**3**). Light yellow powder, ^1^H-NMR (400 MHz, CDCl_3_): *δ* 12.22 (1H, brs, -OH), 10.61 (1H, s, -CHO), 7.66 (1H, d, *J* = 9.6 Hz, H-4), 7.60 (1H, d, *J* = 8.8 Hz, H-5), 6.89 (1H, d, *J* = 8.8 Hz, H-6), 6.33 (1H, d, *J* = 9.6 Hz, H-3), ^13^C-NMR (100 MHz, CDCl_3_): 192.94 (-CHO), 165.51 (C-7), 159.12 (C-2), 156.74 (C-9), 143.36 (C-4), 135.98 (C-5), 114.70 (C-10), 113.42 (C-8), 110.87 (C-3), 108.68 (C-6).

### 4.5. In Vitro BACE1 Enzyme Assay

A BACE1 fluorescence resonance energy transfer (FRET) assay kit (β-secretase, human recombinant) was purchased from PanVera Co. (Madison, WI, USA). The assay was carried out according to the provided manual with slight modifications, as described in a previous study [19]. Quercetin was used as a positive control. The tested concentration range was 0.4–200 µM for **1**–**3** and 2–50 µM for quercetin.

### 4.6. In Vitro ChE Enzyme Assay

The inhibitory activity of the isolated coumarins toward ChE was measured using the spectrophotometric method developed by Ellman et al. [49]. ACh and BCh were used as substrates to assay the inhibition of AChE and BChE, respectively. Each reaction mixture consisted of 140 µL sodium phosphate buffer (pH 8.0), 20 µL of test sample solution, and 20 µL of either AChE or BChE solution, which were then combined and incubated for 15 min at room temperature. **1**–**3** and the positive control (berberine) were dissolved in 10% DMSO. The tested concentration range was 4–250 µM for **1**–**3** and 0.32–20 µM for berberine. Reactions were initiated upon addition of 10 µL of DTNB and 10 µL of either ACh or BCh. Enzymatic hydrolysis, mediated by AChE or BChE, was monitored according to the formation of yellow 5-thio-2-nitrobenzoate anions at 412 nm for 15 min, which were generated by the reaction of DTNB with thiocholine released from ACh or BCh. All reactions were performed in 96-well plates in triplicate, and recorded using a microplate spectrophotometer (Molecular Devices, Sunnyvale, CA, USA).

### 4.7. Kinetics of BACE1 Inhibition

Complementary kinetic methods were employed: Lineweaver–Burk and Dixon plots [50,51]. The mechanism of BACE1 inhibition was evaluated by monitoring the effects of different concentrations of substrate (750, 375, and 250 nM). The test concentrations of **2** in BACE1 inhibition kinetic experiments were 0, 0.4, 2.0, and 10 µM, while those for **3** were 0, 25, 50, and 100 µM. Inhibition constants (*K_i_*) were determined by interpreting the Dixon plot, where the value on the *x*-axis represents *K_i_* [51,52].

### 4.8. Molecular Docking Simulation to Investigate AChE and BACE1 Inhibition Using AutoDock 4.2

Molecular docking was carried out with AutoDock 4.2 program using the default parameters, with slight modification [53]. The target proteins, AChE and BACE1, were obtained from the RCSB Protein Data Bank (PDB, http://www.rcsb.org/), with the respective accession codes 1acj and 2wjo, respectively. The co-crystallized ligands, THA and QUD, were used to generate the grid box for a catalytic inhibition mode. The reported allosteric inhibitors, donepezil and PMF, were also used to compare the interaction residues [24,54], and their 3D structures were downloaded from PubChem Compound (NCBI), with compound CIDs of 3152 and 79,730, respectively. The 3D structures of **1**–**3** were drawn with Chemdraw Ultra 12.0 (CambridgeSoft, Cambridge, MA, USA), and their pK_a_ values were computed at crystallographic pH (pH = 7.5) using the MarvinSketch (v17.1.30, ChemAxon, Budapest, Hungary). Docking simulation of **1**–**3** with AChE and BACE1 were performed individually using AutoDock Tools (ADT), and all torsions were allowed to rotate. The grid box size was 40 × 40 × 40 with a default spacing of 0.375 Å, and the x, y, z, center was 6.312, 71.239, 69.019 for AChE, and 18.167, 36.716, 40.55 for BACE1. The docking parameters were used as defaults of the ADT and Lamarckian genetic algorithm method was employed. The results were analyzed using PyMOL 1.7.4 and Ligplot+.

### 4.9. Inhibition of ONOO^−^-Mediated Protein Tyrosine Nitration

We evaluated ONOO^−^-mediated protein tyrosine nitration using the method of Ali et al. [20], with slight modifications. The tested concentration range of **2** was 12.5–100 µM.

### 4.10. Statistical Analysis

All results are expressed as means ± SEMs of triplicate samples. Results were analyzed using one-way ANOVA and Student’s *t*-test where appropriate (Systat Inc., Evanston, IL, USA). Values of *p* < 0.05 were considered statistically significant.

## 5. Conclusions

The present study revealed that coumarins may have significant anti-AD activity through inhibition of ChEs and BACE1 in the Aβ pathway. In particular, **2**, which has an aldehyde group at 6 position, inhibited BACE1 non-competitively with lower *K_i_* and IC_50_ values compared to **1** and **3**. In addition, the 3D molecular docking study showed that **2** docked into the allosteric site of BACE1. These modeling results were consistent with our in vitro findings, which revealed that inhibitory activities against BACE1 are closely related to the presence of the formyl moiety at position 6 in **2**. Moreover, **2** exhibited potent inhibitory activity against ONOO^−^-mediated tyrosine nitration, which is one of the important therapeutic targets in AD. From the results of the present study, we can conclude that introduction of the formyl moiety to **1** increases the inhibitory activity against ChEs and BACE1, and that its location at position 6 (as in **2**) is more effective that at position 8 (as in **3**). To the best of our knowledge, this is the first report of the potential anti-AD activity of **2** and **3**. Therefore, **2** could play an important role in the development of new therapeutic drugs to treat AD. Further studies are needed to determine if **2** has the same effects in vivo and to clarify the underlying mechanisms.

## Figures and Tables

**Table 1:** Electric eel acethylcholinesterase, horse serum butyrylcholinesterase, and human recombinant BACE1 inhibitory activity of coumarins from *Angelica decursiva*.

Compounds	IC_50_ (µM) ^a^					
	AChE	BChE	SI ^b^	BACE1	*K_i_* Value ^c^	Inhibition Type ^d^
**1**	105.48 ± 0.57	90.14 ± 0.02	0.85	168.54 ± 2.17	-	-
**2**	16.70 ± 1.62	27.90 ± 3.43	1.67	1.31 ± 0.01	2.27	Noncompetitive
**3**	19.13 ± 0.57	87.67 ± 0.48	4.58	39.82 ± 0.31	22.2	Noncompetitive
**Berberine** ^e^	0.14 ± 0.08	9.81 ± 0.35	70.04	-	-	-
**Quercetin** ^e^	-	-	-	22.08 ± 0.57	-	-

^a^ IC_50_ values (μM) were calculated from log dose inhibition curves, and are expressed as means ± SEMs of triplicate experiments. ^b^ Selective index (BChE/AChE) ^c^ BACE1 inhibition constant (*K_i_*) was determined using a Dixon plot. ^d^ BACE1 inhibition type was determined using Dixon and Lineweaver–Burk plots. ^e^ Positive controls. (**-**) not tested.

**Table 2:** Molecular interaction of the acetylcholinesterase active site with **1**–**3** and the inhibitors THA and donepezil.

Compounds	Binding Energy (kcal/mol) ^a^	No. of H-Bond ^b^	H-Bonds Interacting Residues ^c^	van der Waals Interacting Residues ^d^
**1**	−6.3	3	Tyr121, Phe288, Arg289	Trp279, Ile287, Phe290, Phe330, Tyr334
**2**	−8.3	3	Ser200, Tyr334, His440	Ser81, Trp84, Gly118, Glu199, Phe330, Trp432
**3**	−8.0	1	Glu199	Trp84, Gly117, Gly118, Phe330, His440, Gly441
**THA** ^e^	−9.8	1	His440	Tyr442, Phe330, Trp84, Gly118, Trp432, Gly441, Tyr334, Glu199
**Donepezil** ^f^	−10.6	-	-	Tyr70, Ile275, Asp276, Trp279, Ile287, Phe288, Arg289, Tyr334, Tyr121, Ser286, Phe290, Phe330, Phe331

^a^ Binding energy, which indicates binding affinity and capacity for the active site of the AChE enzyme. ^b,c,d^ The number of hydrogen bonds and all amino acid residues involved in the enzyme–inhibitor complex were determined using AutoDock 4.2. ^e,f^ THA and donepezil were used as positive control ligands for catalytic and allosteric sites, respectively.

**Table 3:** Molecular interaction of the BACE1 active site with **1**–**3** and the inhibitors QUD and PMF.

Compounds	Binding Energy (kcal/mol) ^a^	No. of H-Bond ^b^	H-bonds Interacting Residues ^c^	van der Waals Interacting Residues ^d^
**1**	−5.4	1	Asp32	Lys75, Trp76, Val69, Phe108, Ile118
**2**	−7.2	2	Gly13	Ser10, Gly11, Tyr14, Val170, Thr232, Arg307, Pro308, Ala335, Glu339
**3**	−7.0	-	-	Ser10, Gly11, Gly13, Tyr14, Val170, Thr232, Arg307, Ala335, Val336, Glu339
**QUD** ^e^	−9.3	4	Asp228, Asp32, Gly230	Lys107, Lys75, Gly74, Leu30, Thr231, Val69, Tyr198, Ile226, Thr329, Gly34, Arg235, Ser35, Tyr71, Ile118
**PMF** ^f^	−7.8	1	Gly11	Ser10, Tyr14, Thr232, Trp277, Glu303, Gln304, Leu306, Arg307, Pro308, Tyr320, Ala335, Val336, Glu339

^a^ Binding energy, which indicates binding affinity and capacity for the active site of the BACE1 enzyme. ^b,c,d^ The number of hydrogen bond and all amino acid residues involved in the enzyme–inhibitor complex were determined with AutoDock 4.2. ^e,f^ QUD and PMF were used as positive control ligands for catalytic and allosteric sites, respectively.

## Supplementary Materials

The following are available online.
