# Supplementary material for: Kinetics and Molecular Docking Studies of 6-Formyl Umbelliferone Isolated from Angelica decursiva as an Inhibitor of Cholinesterase and BACE1
Source: Molecules. 2017 Sep 24;22(10):1604. doi: 10.3390/molecules22101604 (PMC6151429; doi:10.3390/molecules22101604)
Supplement: Supplementary file 1 [file molecules-22-01604-s001.pdf]

Supplementary Materials: Kinetics and Molecular Docking Studies of 6-Formyl Umbelliferone Isolated from *Angelica decursiva* as an Inhibitor of Cholinesterase and BACE1

Md Yousof Ali[1,#], Su Hui Seong[1,#], Machireddy Rajeshkumar Reddy[2], Sung Yong Seo[2], Jae Sue Choi[1,*], Hyun Ah Jung[3,*]

[1]Department of Food and Life Science, Pukyong National University, Busan 48513, Republic of Korea; yousufbge@gmail.com (M.Y.A.); seongsuhui@naver.com (S.H.S.)
[2]Department of Chemistry, Pukyong National University, Busan 48513, Republic of Korea; rajeshr.chem@gmail.com (M.R.R.); syseo@pknu.ac.kr (S.Y.S.)
[3]Department of Food Science and Human Nutrition, Chonbuk National University, Jeonju 54896, Republic of Korea
[#] These two authors contributed equally to this work
* Correspondence: choijs@pknu.ac.kr (J.S.C.);jungha@jbnu.ac.kr (H.A.J.);
Tel.: +82-51-629-5845 (J.S.C.); Tel.: +82-63-270-4882 (H.A.J.)

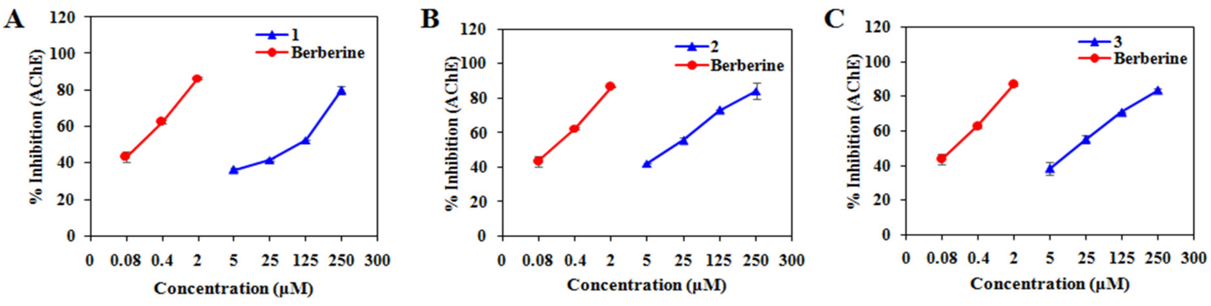

Figure S1. Electric-eel AChE inhibitory activity of **1** (A), **2** (B), and **3** (C). AChE inhibitory activity of **1-3** were evaluated in the concentration range of 5-250 μM. Berberine was used as a positive control. All values are expressed as mean ± SEM of triplicate experiments.

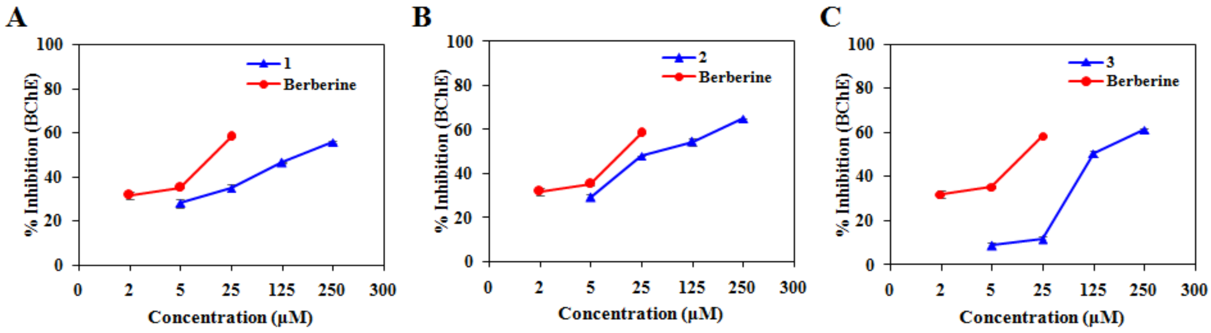

Figure S2. Horse-serum BChE inhibitory activity of **1** (A), **2**(B), and **3** (C). BChE inhibitory activity of **1-3** were evaluated in the concentration range of 5-250 μM. Berberine was used as a positive control. All values are expressed as mean ± SEM of triplicate experiments.

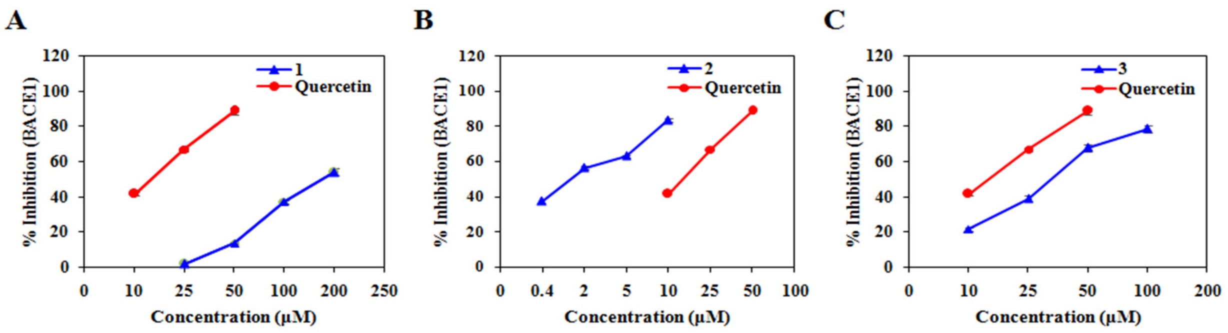

Figure S3. Human recombinant BACE1 inhibitory activity of **1** (A), **2** (B), and **3** (C). BACE1 inhibitory activity of **1**-**3** were evaluated in the concentration range of 0.4-200 μM. Quercetin was used as a positive control. All values are expressed as mean ± SEM of triplicate experiments.
